# An mHealth Application in German Health Care System: Importance of User Participation in the Development Process

**DOI:** 10.1007/s10916-024-02042-6

**Published:** 2024-02-14

**Authors:** Peter Bickmann, Ingo Froböse, Christopher Grieben

**Affiliations:** 1 Institute of Movement Therapy, German Sport University Cologne, Cologne, Germany; 2 University of Applied Management, Ismaning, Germany

**Keywords:** mHealth, User participation, Usability, Health prevention, German health care

## Abstract

This paper addresses the challenges and solutions in developing a holistic prevention mobile health application (mHealth app) for Germany’s healthcare sector. Despite Germany’s lag in healthcare digitalization, the app aims to enhance primary prevention in physical activity, nutrition, and stress management. A significant focus is on user participation and usability to counter the prevalent issue of user attrition in mHealth applications, as described by Eysenbach’s ‘law of attrition’. The development process, conducted in a scientific and university context, faces constraints like limited budgets and external service providers. The study firstly presents the structure and functionality of the app for people with statutory health insurance in Germany and secondly the implementation of user participation through a usability study. User participation is executed via usability tests, particularly the think-aloud method, where users verbalize their thoughts while using the app. This approach has proven effective in identifying and resolving usability issues, although some user feedback could not be implemented due to cost-benefit considerations. The implementation of this study into the development process was able to show that user participation, facilitated by methods like think-aloud, is vital for developing mHealth apps. Especially in health prevention, where long-term engagement is a challenge. The findings highlight the importance of allocating time and resources for user participation in the development of mHealth applications.

**Supplementary Information:**

The online version contains supplementary material available at 10.1007/s10916-024-02042-6.

## Introduction

Germany lags far behind comparably developed welfare states when it comes to digitalization of the healthcare sector [1]. For this reason, the current government wants to push ahead with digitalization [2]. Responsibility however for the digitalization lies largely with the service providers and healthcare insurances, which enjoy particular autonomy in Germany due to their self-governance rights [1]. In addition, a shortage of skilled workers and an ageing population are forcing those responsible to take action [3, 4]. Holistic preventive healthcare must play an important role in solving these problems in order to relieve the burden on the healthcare sector in long term through primary prevention. Digital resources like mobile health applications (mHealth apps) are necessary to relieve and expand the conventional health care system and play a part in this primary prevention [5, 6]. On behalf of a German health insurance company an attempt is being made to develop a holistic prevention app, the *Digital Health Companion* (DHC), in a multidisciplinary team. The DHC aims to help people regarding a health-oriented lifestyle in the areas of physical activity, nutrition, and stress management. According to the prevention guidelines from Germany, these are three of the four most important areas in terms of sustainable prevention [7]. The presentation of the app in a scientific context and its functionality will therefore form part of this paper.

However, mHealth applications are facing the extreme challenge of user attrition. Eysenbach coined the term ‘law of attrition’ here [8]. The rule states that after a certain period of time, only so called ‘hardcore users’ regularly take part in mHealth measures. These make up only a small percentage of all users. The majority no longer uses mHealth after a while due to relatively low motivation or face usability problems [8]. To ensure high effectiveness, this drop-out rate should be as low as possible. In addition to a team of experts, it is also essential to involve potential users in the development process of an mHealth application. This is referred to as user participation in the development process [9]. User participation can have positive influence on usage behavior and can therefore be a positive part of the whole development process [10]. This paper presents user participation in form of a usability study [10, 11] as well as a short questionnaire on intrinsic motivation of using the app [12].

The development process takes place in a scientific and university context and is therefore, as so often, confronted with major obstacles such as limited budget or external service providers. This paper therefore aims for two objectives: Firstly, the structure and functionality of an mHealth application for the German healthcare sector is presented. Secondly, the implementation of user participation in the form of a usability study is explained and placed in the context of the overall development in a multidisciplinary team. To this end, the concept of user participation and the meaning of usability in this context will be explained in more detail. The development process and the functionality of the app are then presented, followed by the usability study. Finally, the results are put into context with the development process and possible conflicts between the developers’ (experts) and users’ perspectives are discussed.

## User Participation and Usability

As stated, the loss of users is one of the biggest hurdles for mHealth applications [8]. In addition to a lack of relatively low user motivation, usability problems are particularly common reasons [13]. To minimize these, user participation is becoming increasingly important in the development process [14]. Studies show that involving users in the development process of mHealth applications is crucial to ensure that they meet the needs and expectations of end users and thus achieve greater acceptance and effectiveness [15, 16].

A possibility to involve users are usability tests during the app development process [10, 12]. In the critically important field of health prevention, where engaging the target population poses significant challenges [17, 18], testing and refinment of usability is paramount [19]. The high contextuality of mHealth interventions prevents the exact transferability of results from comparable studies in this topic and must be carried out individually [19, 20] Consequently, usability testing, regardless of the results, should be considered valuable [21, 22] and involving the potential target group in the development process can increase acceptance of mHealth apps [19, 23]. No matter how well informed experts are about a certain topic, the integration of the target group into the development process is inevitable [12]. Various studies have already shown that usability testing of mHealth apps can contribute not only to an improvement of usability itself, but also in content of the app [22, 24]. As in comparable studies, the think-aloud method (TA) was mainly used here to investigate usability [12]. In this method, users speak their thoughts out loud at any time during app usage and are recorded. The video and audio materials are then analyzed.

## Methods

This section first presents the basic development process and the main functions of the DHC. Then the implementation of the usability study as part of the user participation is described.

### Development Process and Application Features

Getting more people engaged in an active lifestyle through health prevention can be difficult. Demands and requirements differ between target groups [25, 26]. Barriers like lack of motivation to make lifestyle changes or lack of an overview of programs can also make an implementation more difficult [26]. An app must convince not only through its content, but also through its structure and interface. The DHC was therefore developed from a team of experts in the fields of physical activity, nutrition and stress management with the support of UX designers as well as programmers. The app is designed to make health prevention as low-threshold as possible, independent of users’ time and location. To this end, the first impression of the app must be kept very simple [27]. Users should receive an individual result after answering only a few questions concerning their health. Therefore, based on existing questionnaires, four multiple choice questions were developed for each of the three prevention areas using standardized questionnaires from research. These questionnaires were the EHIS-PAQ [28] and GPAQ [29] (physical activity), the Intuitive Eating Scale-2 [30] (nutrition) and PSQ-20 (stress management) [31]. The resulting questionnaires, the so-called Quick Checks (QC), used in the DHC can be found in supplementary material 1. Through the results of this entry test users set a first health goal, which they then specify with the help of a digital coach (chatbot).

**Fig. 1:**
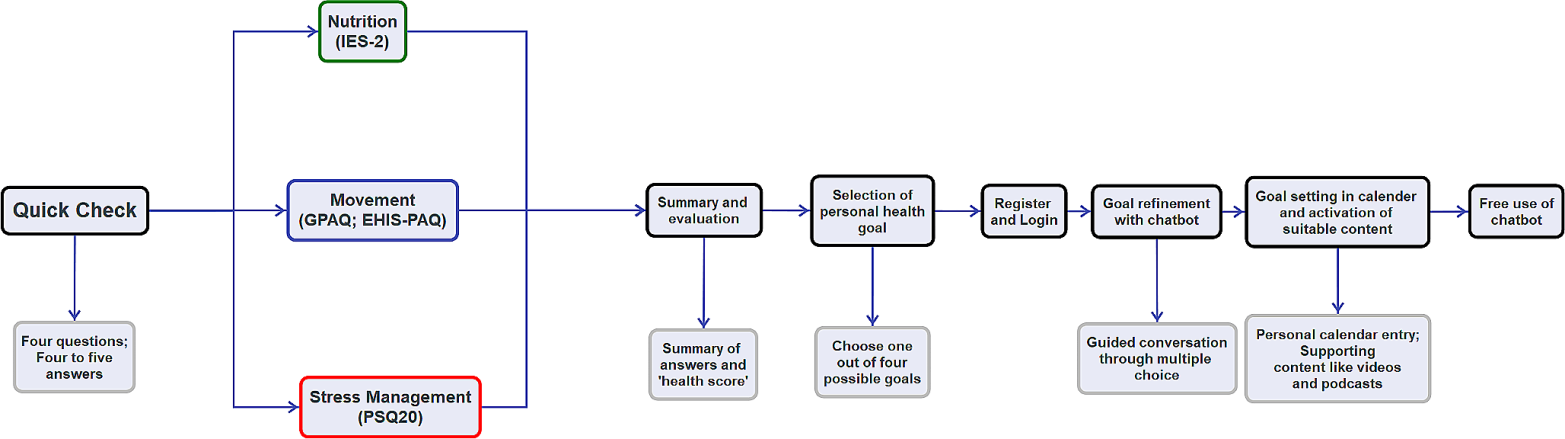
Overview of the app structure and main functions

After completing the QC, users can choose between a maximum of four individual health goals to implement health prevention into daily life. The selection of goals depends on the answers in the QC. Additionally, a short summery of given answers is created. This makes it easier for the user to set their answers in context and can help to form an intention, i.e. to change something about their own health behavior [32]. After this, the login takes place. With support of a chatbot called ‘Ben’, the user refines the personal goal to match it exactly to the daily routine, for example by choosing weekdays for a push-up reminder. Based on the social cognitive theory, these specific goals are intended to help users change their own health behavior in long term [33]. As a support to the user, different media content (videos, podcasts, blogs, GIFs) is made available in personal library. Interim goals such as small exercises at the workplace are transferred into the app-calendar. An overview of the schematic structure and features of the app is provided by Fig. 1.

**Fig. 2:**
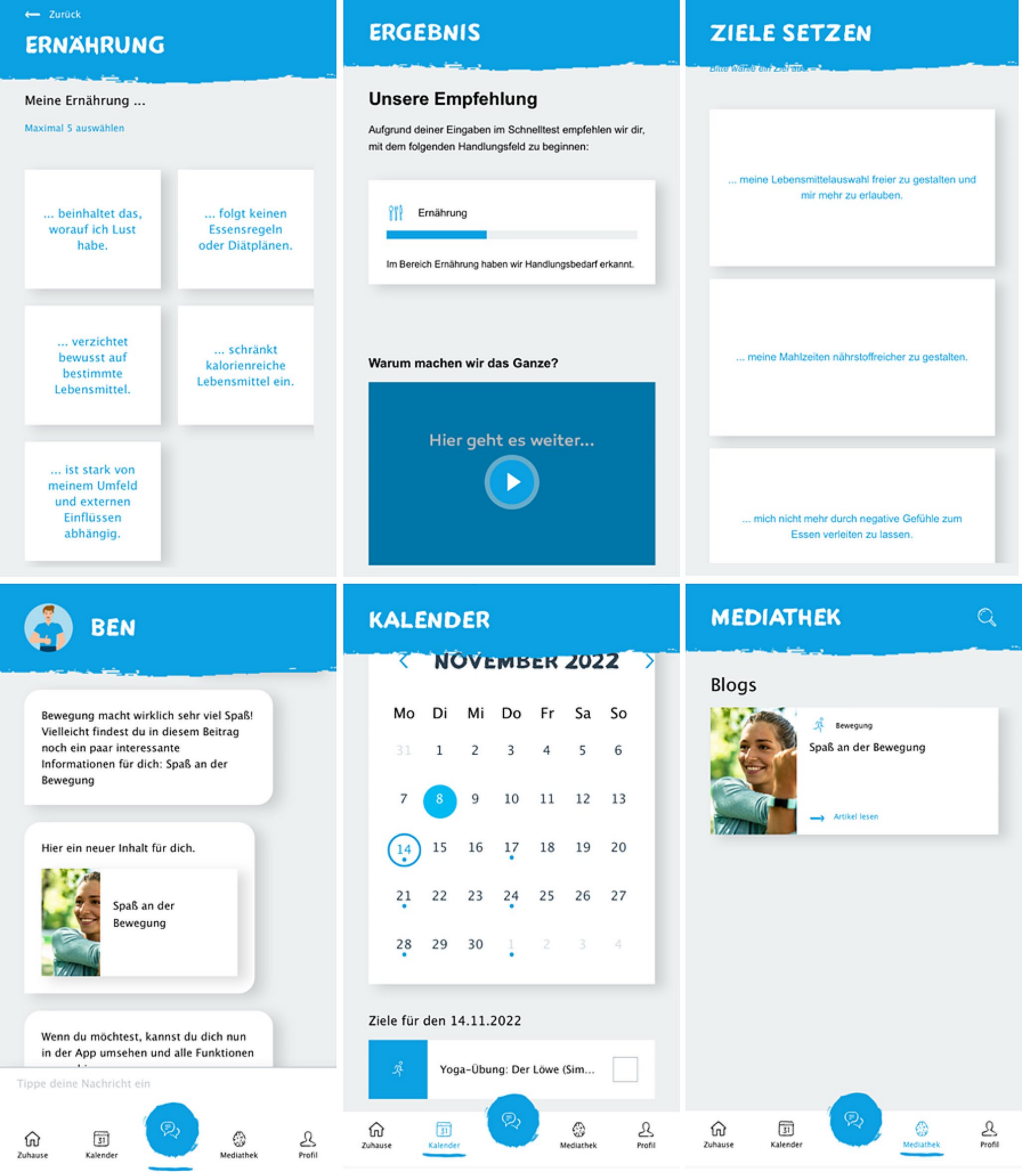
Main functions of the application (from top left to bottom right): Quick Check; Result Screen; Health Goal Selection; Health Coach (chatbot); User Calendar; Media Library

Screenshots of the app’s main functions are additionally shown in Fig. 2. Since the app is currently only available in German, the free translations into English for all three different quick checks can also be obtained in Supplementary material 1.

The chatbot can also be consulted on prevention issues at any time. In further steps of the technical development, the users’ questions will be answered by health experts in the first time period after the release of the app. The experts’ answers are then programmed as automatic conversations and gradually integrated into the app. The DHC should thus offer the user maximum personal benefit and be integrated into everyday life as low threshold as possible.

To achieve this goal, the operation and usability of the app must be made as simple as possible. Therefore, evaluating usability among the target audience is vital to the app’s success.

### Recruitment and Participants for the Usability Test

Participants in this study had to have statutory health insurance in Germany, as the app was developed specifically for these people. They were recruited via personal contacts and did not receive any incentives for participating. A sample with good diversity in terms of age and educational status was selected to correspond approximately to insured persons of the commissioning health insurance. Eleven people (seven male) participated in this usability study. Average age was 45.55 ± 18.44 (min. 22; max. 83) years. Five participants had university degrees, two a high school diploma and four middle maturity or lower.

## Materials

Basically, the participants could use all functions of the app. However, the chatbot could not yet respond to new questions from the participants and some media content as well as some graphics were still missing. In addition, various bugs occurred and there could be isolated crashes. For using the app all participant used the same iPhone provided by the developer which they could hold freely in their hands. The smartphone was connected to a notebook and screen as well as participants voice were recorded.

Questionnaires on personal data, usability and intrinsic motivation to use the app were answered digitally.

Regarding usability the System Usability Scale (SUS) [34] was used. A short form of the Intrinsic Motivation scale (IMI) for measurement of intrinsic motivation [35].

The full questionnaire in German and freely translated to English can be found in Supplementary material 2.

### Design

At start, participants were informed on the general process of the study and contents of the app and recording of their voice. They filled out the questionnaire on demographics, personal experience and general usage of mHealth applications. After that, TA was explained and participants performed a practice task using a weather app like in comparable studies [12]. Participants were informed that the experimenter would only interrupt if a pause in speech lasted more than five seconds [36]. To use the app, participants were given a list of eleven tasks in total, which they had to complete. The list can be found in Supplementary material 3.

Required time for each task was measured. During the tasks, participants were asked to share their thoughts about the individual tasks, but also about the app interface [24]. After the last task, participants answered the SUS and IMI.

### Data Collection and Analysis

Data collection and analysis was guided by similar studies [12, 24, 37, 38]. After completion of the test each task was rated from 0 to 3 [37]:

- 0: Task was not completed.
- 1: Task was completed with physical assistance.
- 2: Task was completed with verbal assistance.
- 3: Task was completed without assistance.

The usability problems encountered while using the app were first recorded, categorized and then rated by the investigators according to their severity using the Nielsen severity scale [39] (Table 1).

Watching and listening multiple times to screen and audio recordings, author A and a research assistant extracted qualitative comments. The evaluation was carried out independently and blinded. Discrepancies were discussed together in the final evaluation step. If no agreement could be reached, author C was consulted. The user comments were then related either to perceived usefulness [24] or the individual tasks [37].

### Descriptive Results of the Usability Tests

On a five-point scale participants rated their familiarity with mHealth apps on average with 2.0 and described the frequency of use as ‘infrequent’.

**Fig. 3:**
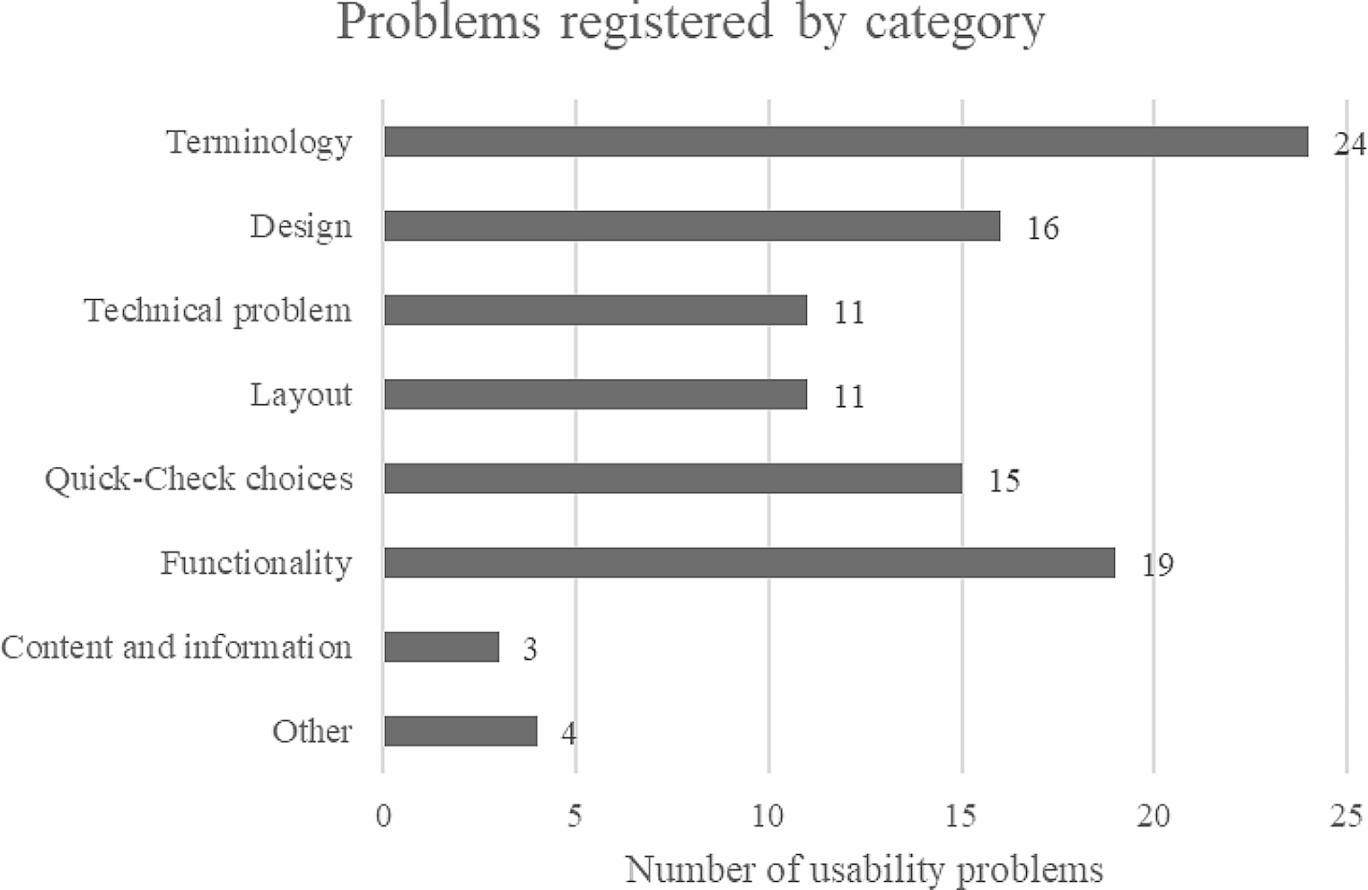
Usability problems registered sorted by category

**Fig. 4:**
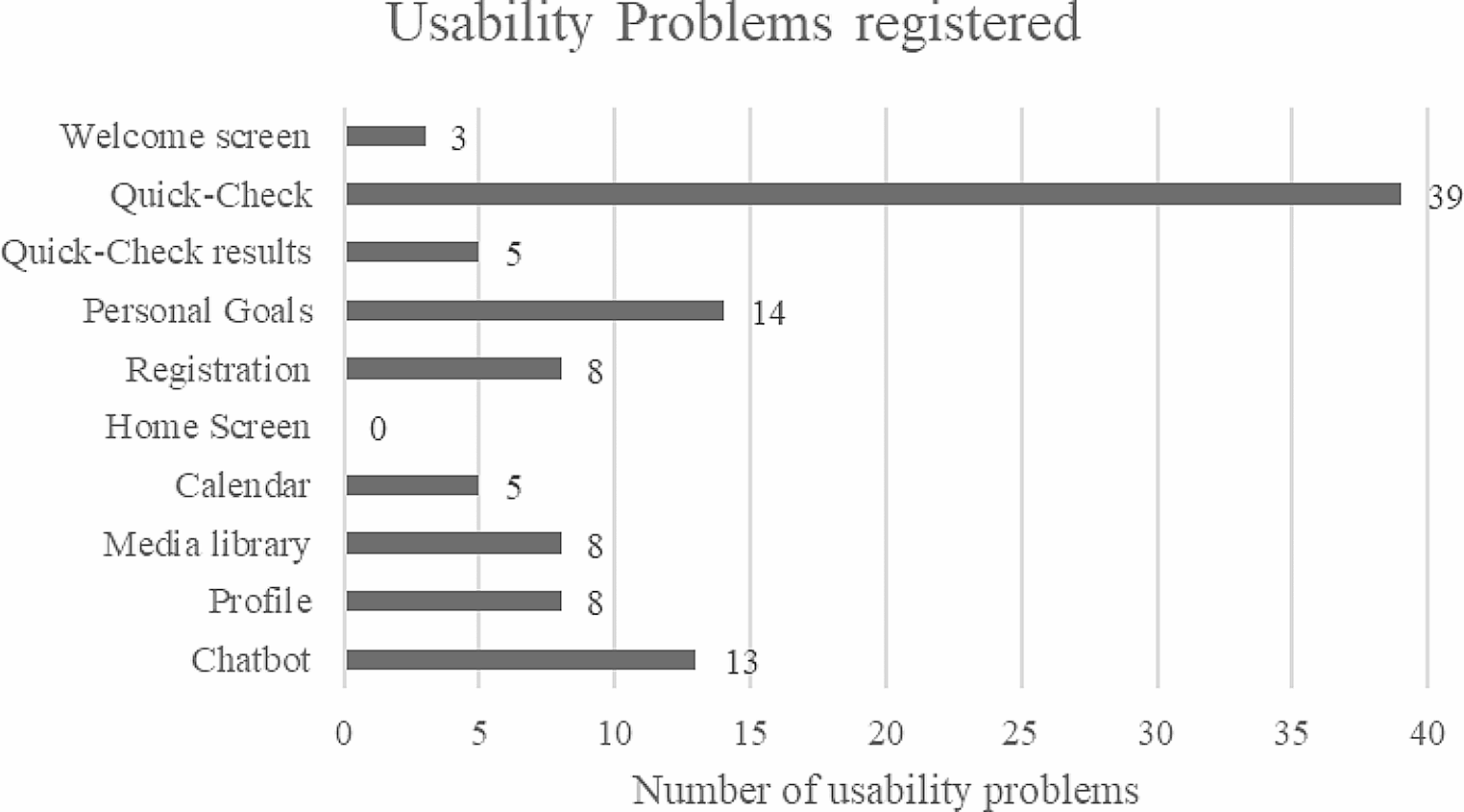
Usability problems registered sorted chronologically by usage of the app

A total of 103 usability problems were registered, 9.36 ± 4.12 per participant. These usability problems were sorted by category (Fig. 3):

Chronologically sorted by the structure of the app for a user while using it, the following Fig. 4 shows the parts of the app where the problems occurred:

Registered usability problems were rated according to Nielsen severity scale (Table 1):

**Table 1:** Registered usability problems rated according to Nielsen severity scale

Rating	Definition	Reg. Problems
0	I don’t agree that this is a usability problem at all.	1
1	Cosmetic problem only: need not be fixed unless extra time is available on project.	17
2	Minor usability problem: fixing this should be given low priority.	49
3	Major usability problem: important to fix, so should be given high priority.	27
4	Usability catastrophe: imperative to fix this before product can be released.	9

Examples for a rating of 4 were no suitable selection option in the QC or no indication to swipe left to see more options regarding user’s body shape.

### Task Performance

To complete each of the tasks, participants on average needed 17.7 ± 4.44 (min: 11.01; max: 26.97) minutes. Detailed information here can be found in supplementary material 3.

**Fig. 5:**
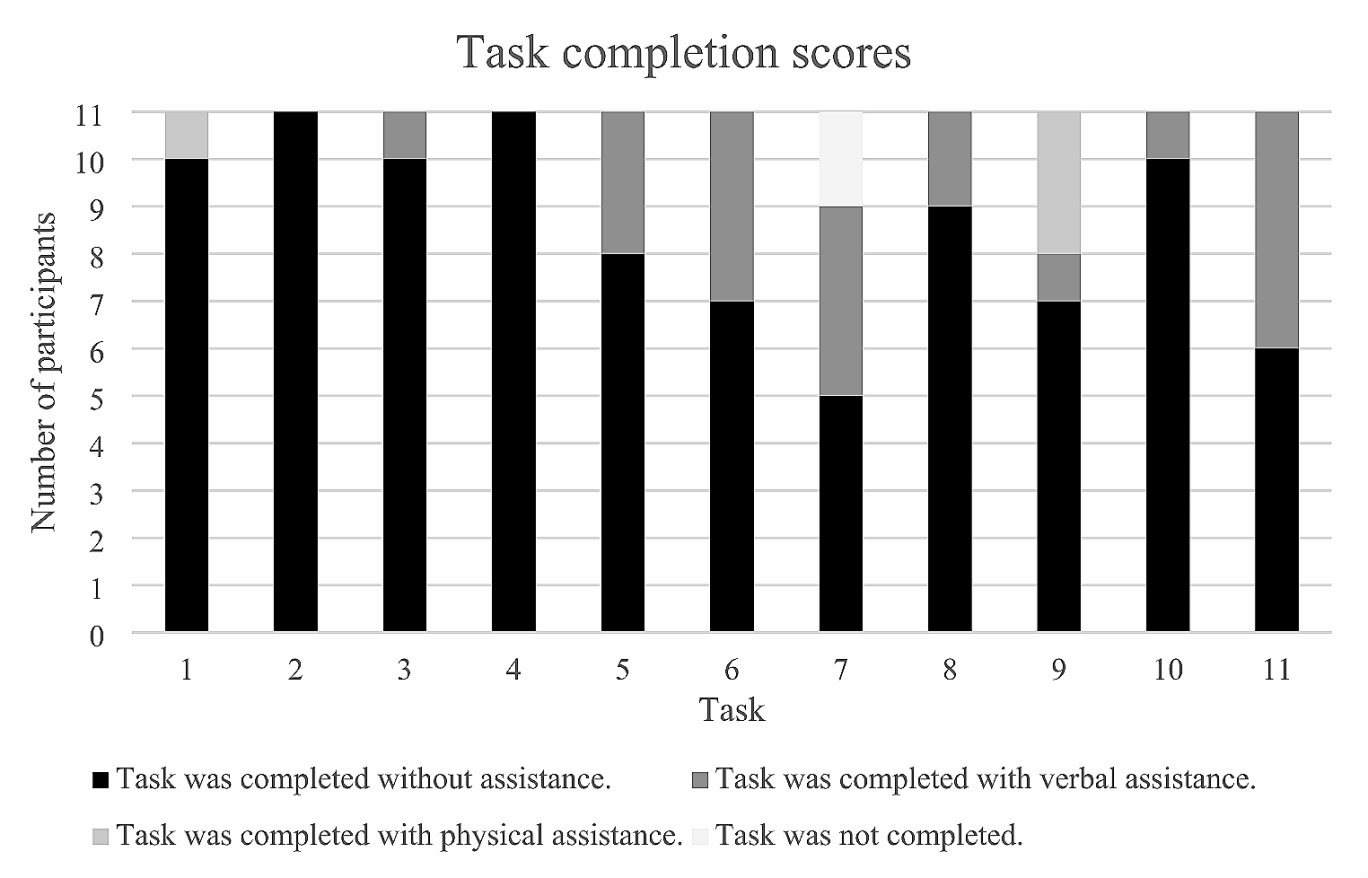
Participants’ performance in each of the user tasks

The participants’ performance for each of the eleven tasks is presented in Fig. 5:

### System Usability Scale and Intrinsic Motivation

The SUS consists of ten questions with a rating from 1 (very low usability) to 5 (very high usability) for each question (supplementary material 2). A conversion results in an overall rating between 0 and 100 points. A final rating above 68 points is considered above average and a fine usability [34]. On the SUS in this study, participants on average rated the usability 82.05 ± 11.77 (median: 85.00; min: 62.50; max: 97.50).

The short form of the IMI was used for self-reported evaluation of how much the participants valued the DHC. Seven questions were answered using a seven-point scale. The mean IMI score was 5.06 ± 1.51 (median: 5.57; min: 3.00; max: 7.00).

### Connections and Correlations

Table 2 gives an overview of connections and correlations (Spearman) between different factors measured in this study.

**Table 2:** Correlation (Spearman) between different factors measured in this usability study

Factor 1	Factor 2	r-value	*p*-value
mHealth experience	Task performance	0.537	0.089
mHealth experience	Number of usability problems	− 0.158	0.642
Usage of mHealth apps	Task performance	**0.623**	0.041*
Usage of mHealth apps	Number of usability problems	− 0.014	0.966
Age	Number of usability problems	− 0.304	0.363
Age	Task performance	**− 0.812**	0.002*
Completion time	Number of usability problems	0.475	0.140
Completion time	Task performance	0.183	0.590
SUS score	Task performance	0.554	0.077
SUS score	Number of usability problems	− 0.110	0.747
SUS score	Age	− 0.128	0.707
IMI score	Task performance	0.414	0.206
IMI score	Number of usability problems	− 0.509	0.110
IMI score	Age	− 0.256	0.447

*Significant correlation; *p* < 0.05

### Qualitative Assessment

Overall, data analysis revealed a certain discrepancy between the perceived performance of the app and participants’ verbal statements. Despite the usability problems and difficulties with the tasks, participants on average were very positive about the app. They reported the app was easy to handle, simple to use and clearly designed. This discrepancy is particularly noticeable in task completion. None of the participants expressed problems with the tasks, while some of them did not complete them.

An example is task 7, where participants were asked to change their password. To change the password, users must press the edit button for personal data in the profile screen. This task had the lowest completion score. Participants’ sample sentences were for example: ‘I don’t know what to do here, I only see a button for logout’ or ‘I must be wrong here, I will go to the home screen’. After the edit button was shown to them, sentences like ‘Ah, there it is!’ were made. Additionally, a participant who changed password without problems said: ‘I suppose that many have a problem finding this button.’

Still no participant mentioned problems or difficulties after the final task completion in overall feedback but commended various features of the app.

## Discussion and Benefits of User Participation

### Principal Findings

In this study, TA with task completion was used to analyze usability of a self-developed holistic health prevention app for user participation in the development process by a multidisciplinary team. Eleven participants were asked to complete eleven tasks in the app. Overall, 103 usability problems were reported of which nine were rated as usability catastrophe, 27 as a major and 49 as minor problems. Most problems were related to terminology, functionality, design, and QC. Regarding contents of the app, 39 problems were reported doing QC and 14 while choosing individual health goals. These results are integrated into the development process to improve the quality of the app and adapt it to the needs of potential users.

Overall participants rated the usability of the app highly in their statements and in responding to the SUS. Regarding participants’ task completion, a significant correlation was found for self-reported usage of mHealth apps and a negative correlation for participants’ age.

### App Customization Through User Participation

The aim of the DHC is to provide users with an easy and short introduction and to set an initial individual health goal after just a few questions (QC). These questions are based on the content of scientific health questionnaires (see supplementary material 1). Scientific questionnaires were used as a basic framework. In order for users to be more likely to answer these questions, they had to be shortened considerably [40]. Shortening can have an enormous impact on the significance and can cause problems [41, 42]. Next to scientific quality criteria important information can be lost or the questionnaire may not cover all relevant aspects of a specific topic.

Problems of this type were also observed with striking frequency in this study. Nearly 36.8% of all usability problems occurred during QC, which was only one of the tasks. Since QC is the first function and has a decisive influence on the first overall impression [43], working on the problems is of high priority. Problems related mostly to terminology and choices. In terms of understanding, one user did not understand an answer option (‘What is brain exercises’). In the further process, alternatives were developed for the answer option ‘brain exercise’. The new options included ‘gaming’, ‘puzzles’ or ‘active relaxation’ and must be evaluated again. One did not fully understand a question: ‘Does this question refer only to my private life or also to my work?’ As a result, a short note was added to each question if it related exclusively to work or leisure time. Also the fifths response option for nutrition was not perceived which resulted in non-sufficient answers in some cases: ‘For me none of the answers fit.’ or ‘I think all options are bad for me.’ The reason was that only four answer options were visible on the screen and you had to scroll down for the fifth. As seen in Fig. 2 (top left), this was fixed by a programmer. As mentioned, changing the password (task 7) was a problem for some participants. The symbol (pen writing on paper) was then significantly enlarged and placed more centrally.

A very important part of the app is the individually displayed content for each user (videos, audios, GIFs). As the scripts were written by experts in the respective field with the support of a content producer, it was very important to review this content by potential users. All content played out was perceived as understandable and appropriate by the users. One user recommended videos with real people instead of animated ones. This point leads to a problem regarding user participation in the development process.

Involving potential user into the development of an mHealth app as early as possible is important and brings many benefits [44], but also conflicts can occur here. One example is the conflict between user perspective and evidence [45]. Regarding the DHC, it is not possible to just use recommendations for the QC from participants. The funder claims that this app is based on scientific instruments. The answers in the QC must therefore at least always be based on existing questionnaires or be similar. In addition, the costs and benefits must always be weighed up during the development. Users do not have to take this into account [46]. This can lead to problems, especially for such a project with a limited budget in a scientific context. One example of this study concerns the font size of the app, which was rated as too small in some cases. Since a change in the font size by the programmer would have required partial changes to the design, a new budget for UX designers would have been necessary. This was no longer planned at that time. In such cases, the costs and benefits must be weighed up on individual basis. In this case, the decision was made not to make changes, as only one person commented on this and a change in font size can also be set individually depending on the smartphone.

This shows how important user participation is in a development process and, depending on the circumstances, as early as possible. However, this feedback should never be accepted blindly. Various factors such as evidence or costs/benefits must always be weighed up.

### Demand Characteristics in Think Aloud and Further Limitations

Several differences occurred regarding the participants’ positive awareness of the app and the number of usability problems. One reason could be the principle of demand characteristics [47]. In this TA study, the experimenter sat directly behind the participants. The participants were also explained that the experimenter would intervene after five seconds of silence. In order to make the experimenter feel as positive as possible regarding the app, it is possible that the participants rated the app rather more positively at the end. Furthermore, there could be difference in the general impression of the app and the processing of the individual tasks. Participants may not be aware of the usability problems expressed or may not rate them as particularly serious themselves.

The number of participants appears small, but is reasonable compared to similar studies [12, 37]. Participants had to have statutory health insurance in Germany and an attempt was made to design the test group as heterogenous as possible regarding different criteria like age, gender or educational status. Furthermore, it can be assumed that three quarters of all usability problems of an application can already be found by testing with five participants [48].

The experience of participants in using mHealth apps is generally considered to be rather low. The possible unbiasedness of the subjects may be a strength of the study in this regard. On the other hand, hardly any assessments are possible by the participants regarding previous experiences with other apps.

In addition, there are general limitations of TA. It is based on the assumption that participants can verbalize the contents of their working memory [49]. This situation is quite unusual for people. The pressure of acting and talking at the same time can cause unwanted statements and reactions from the participants [50].

## Conclusions

User participation is a must have in mHealth development. Communication of health issues is difficult and user feedback is therefore all the more crucial. This paper presents the development and functionality of a holistic prevention app for people with statutory health insurance in Germany. The usability of this app was tested using TA at an early stage of development. Important problems such as the clarity of symbols or the comprehensibility of technical terms were identified regarding usability and solved. However, a few comments could not be implemented. Weighing up evidence and costs/benefits, especially in the area of health prevention, is very important. In addition, TA has proven to be a profitable method for identifying usability problems. Time and personnel resources for user participation should therefore be included in the calculation of mHealth apps before and during development. For mHealth, where only ‘hardcore users’ use a product in the long term, user centricity is one of the most important success factors.

## Electronic Supplementary Material

Below is the link to the electronic supplementary material.

Supplementary Material 1

Supplementary Material 2

Supplementary Material 3

## Data Availability

The data that support the findings of this study are available from the corresponding author upon reasonable request.

## Abbreviations

DHC: Digital Health Companion (the developed mHealth application)
IMI: Intrinsic Motivation Inventory
mHealth: Mobile Health
QC: Quick Check (DHC introduction test)
SUS: System Usability Scale
TA: Think aloud method
