# Supplementary material for: An mHealth Application in German Health Care System: Importance of User Participation in the Development Process
Source: J Med Syst. 2024 Feb 14;48(1):20. doi: 10.1007/s10916-024-02042-6 (PMC10866790; doi:10.1007/s10916-024-02042-6)
Supplement: Supplementary file 1 — Supplementary Material 1 [file 10916_2024_2042_MOESM1_ESM.docx]

**Answers and first health goals from the quick check of the DHC**

**Nutrition Questions:**

**Q1:** My diet … (maximum 5 answers)

**A1:** … contains what I feel like.

**A2:** … dies not follow any food rules or diet plans.

**A3:** … consciously avoids certain foods.

**A4:** … restricts high-calorie foods.

**A5:** … is strongly dependent on my environment and external influences.

**Q2:** I listen to the fact that my body … (maximum 4 answers)

**A1:** … tells me what to eat.

**A2:** … tells me when to eat.

**A3:** … tells me how much to eat.

**A4:** … regulates my diet by hunger and satiety.

**A5:** None of the above.

**Q3:** To satisfy my hunger … (maximum 5 answers)

**A1:** … I usually crave healthy foods.

**A2:** … I eat foods that give me energy for a long time.

**A3:** … I eat what is available at the moment without paying attention to the ingredients.

**A4:** … I like to buy something on the go.

**A5:** … I often resort to ready-made products.

**Q4:** I eat … (maximum 4 answers)

**A1:** … when I am emotionally upset.

**A2:** … out of boredom.

**A3:** … when I am lonely and/or stressed.

**A4:** … even when I am not hungry.

**A5:** None of the above.

**Nutrition First Health Goals:**

Based on the choices above, a user will receive a maximum of 4 individual health goals, which one of them will be individualized and specialized in further usage of the application with the chatbot. Four each of the four questions above, one of the following goals may be possible to select:

**G1:** My goal is …

- … to make my food choices more freely and allow myself to eat more.
- … to make my diet more self-determined and individual.
- … to make my food choices more freely and to adapt them to my needs and preferences.
- … to make my food choices even more freely and learn to trust my intuition.
- … to adapt my diet to my needs and preferences and to trust my intuition.

**G2:** My goal is …

- … to listen more to my feelings of hunger and satiety.
- … to better perceive and satisfy the needs of my body.
- … to better distinguish appetite and hunger.
- … to better recognize my body's signals of satiety.

**G3:** My goal is …

- … to consciously deal with my food choices in order to do something good for my body.
- … to make my meals more nutritious.
- … to get to know healthy alternatives for in between and to integrate them into my everyday life.
- … to make my meals more nutritious and to integrate simple alternatives into my everyday life.
- … to get to know healthy alternatives and integrate them into my everyday life.
- … to be more aware of my diet and to balance it in the long term.
- … give myself more time for my diet.
- … to take more time for my diet in order to make it more conscious and balanced.
- … to integrate more good alternatives into my everyday life and to be more aware of my diet.

**G4:** My goal is …

- … to no longer be tempted to eat by negative feelings.
- … to see food intake as a source of energy for my body.
- … to listen to the physical signals of my feeling of hunger.
- … to learn an alternative coping strategy to eating.

**Physical Activity Questions**

**Q1:** Describe your predominant occupation. (maximum 1 answer)

**A1:** Sitting

**A2:** Physically hard

**A3:** Standing

**A4:** Walking

**Q2:** Describe how you get from place to place each day. (maximum 2 answers)

**A1:** Motorized

**A2:** Public transport

**A3:** On foot

**A4:** Active mode of transport

**Q3:** Describe your most common recreational activity(ies) on a daily basis. (maximum 2 answers)

**A1:** Put your feet up

**A2:** Brain exercises

**A3:** Active

**A4:** House & garden work

**Q4:** Describe your weekly sports activity. (maximum 1 answer)

**A1:** None

**A2:** Irregular

**A3:** Daily

**A4:** Regular

**Physical Activity First Health Goals:**

Based on the choices above, a user will receive a maximum of 3 individual health goals (the area of sports is not included yet), which one of them will be individualized and specialized in further usage of the application with the chatbot. Four three out of the four questions above, one of the following goals may be possible to select:

**G1:** My goal is …

- … to find a balance to my physically demanding work.
- … to find a balance to my monotonous standing work.
- … to find a balance to my mainly walking work.

**G2:** My goal is …

- … to find an active way to get from place to place.
- … to travel less by bus and train and to find an active way to get from place to place.
- … to make routes to be covered more varied and even more active.

**G3:** My goal is …

- … to find an active balance to recharge your batteries.
- … to find another hobby that is fun and more active.
- … to find another hobby that I enjoy and is more actively challenging.

**Resource Management / Stress Management Questions**

**Q1:** I have … (maximum 4 answers)

**A1:** … little worries.

**A2:** … tangible goals.

**A3:** … fears for the future.

**A4:** … problems that overwhelm me.

**Q2:** I feel … (maximum 4 answers)

**A1:** … calm.

**A2:** … balanced.

**A3:** … exhausted.

**A4:** … tense.

**Q3:** My everyday life includes … (maximum 4 answers)

**A1:** … enough time for me.

**A2:** … suitable requirements.

**A3:** … deadline pressure.

**A4:** … too many tasks.

**Q4:** I am … (maximum 4 answers)

**A1:** … full of energy.

**A2:** … in a safe environment.

**A3:** … often listless.

**A4:** … rarely fun to be around.

**Resource Management / Stress Management First Health Goals:**

Based on the choices above, a user will receive a maximum of 4 individual health goals, which one of them will be individualized and specialized in further usage of the application with the chatbot. Four each of the four questions above, one of the following goals may be possible to select:

**G1:** My goal is …

- … to develop a positive attitude towards challenges.

**G2:** My goal is …

- … to create a balance through physical relaxation.

**G3:** My goal is …

- … to cope with arising requirements without any worries.

**G4:** My goal is …

- … to fill my everyday life with things that bring me joy and give me energy.
