# Supplementary material for: An mHealth Application in German Health Care System: Importance of User Participation in the Development Process
Source: J Med Syst. 2024 Feb 14;48(1):20. doi: 10.1007/s10916-024-02042-6 (PMC10866790; doi:10.1007/s10916-024-02042-6)
Supplement: Supplementary file 2 — Supplementary Material 2 [file 10916_2024_2042_MOESM2_ESM.docx]

**Freely translated Questionnaire**

**Personal Data**

1. How old are you? _____years
2. To which gender do you assign yourself?
3. What is your highest educational attainment?
4. What is your current body weight? _____kg
5. How tall are you? _____cm
6. How comfortable do you feel using health apps?
   1. Not familiar at all
   2. A little bit
   3. So-so
   4. Good
   5. Completely familiar
7. How often do you use health apps?
   1. Never
   2. Rarely
   3. Several times a month
   4. Several times a week
   5. Daily

**System Usability Scale**

Answers range from 1 (= I do not agree at all.) to 5 (= I fully agree.).

1. I think I would like to use the app frequently.
2. I found the app unnecessarily complex.
3. I found the app easy to use.
4. I think I would need the help of a tech savvy person to use the app.
5. I thought the various features of the app were well integrated.
6. I think the app contains too many inconsistencies.
7. I imagine most people will learn how to use this app very quickly.
8. I found the app very cumbersome to use.
9. I felt very safe using the app.
10. I had to learn a lot before I could start using the app.

**Intrinsic Motivation Inventory**

Answers range from 1 (= I do not agree at all.) to 7 (= I fully agree.).

1. I think I could benefit from using this app.
2. I think that the use of this app is useful.
3. I think the use of this app is important.
4. I would be willing to use this app again as it is valuable to me.
5. I think this app can help me overall.
6. I think using this app is beneficial for me.
7. I think that this app is important.

**Original Questionnaire in German Language**

**Personenbezogene Daten**

1. Wie alt sind Sie? _____Jahre
2. Welchem Geschlecht ordnen Sie sich zu?
3. Welchen höchsten Bildungsabschluss haben Sie?
4. Wie ist Ihr aktuelles Körpergewicht? _____kg
5. Wie groß sind Sie? _____cm
6. Wie vertraut fühlen Sie sich im Umgang mit Gesundheitapps?
   1. Gar nicht vertraut
   2. Ein wenig
   3. Geht so
   4. Gut
   5. Vollkommen vertraut
7. Wie häufig nutzen Sie Gesundheitsapps?
   1. Nie
   2. Selten
   3. Mehrmals monatlich
   4. Mehrmals wöchentlich
   5. Täglich

**System Usability Scale**

Die Antwortskala reicht von 1 (= Ich stimme überhaupt nicht zu.) bis 5 (= Ich stimme voll zu.)

1. Ich denke, dass ich die App gerne häufig benutzen würde.
2. Ich fand die App unnötig komplex.
3. Ich fand die App einfach zu benutzen.
4. Ich glaube, ich würde die Hilfe einer technisch versierten Person benötigen, um die App benutzen zu können.
5. Ich fand, die verschiedenen Funktionen der App waren gut integriert.
6. Ich denke, die App enthält zu viele Inkonsistenten.
7. Ich kann mir vorstellen, dass die meisten Menschen den Umgang mit dieser App sehr schnell lernen.
8. Ich fand die App sehr umständlich zu nutzen.
9. Ich fühlte mich bei der Benutzung der App sehr sicher.
10. Ich musste eine Menge lernen, bevor ich anfangen konnte, die App zu verwenden.

**Intrinsic Motivation Inventory**

Die Antwortskala reicht von 1 (= Ich stimme überhaupt nicht zu.) bis 7 (= Ich stimme voll zu.)

1. Ich denke, ich könnte von der Nutzung dieser App profitieren.
2. Ich denke, dass die Nutzung dieser App nützlich ist.
3. Ich denke, die Verwendung dieser App ist wichtig.
4. Ich wäre bereit, diese App erneut zu verwenden, da sie für mich wertvoll ist.
5. Ich denke, diese App kann mir insgesamt helfen.
6. Ich denke, die Verwendung dieser App ist für mich von Vorteil.
7. Ich denke, dass diese App wichtig ist.
