# Supplementary material for: An mHealth Application in German Health Care System: Importance of User Participation in the Development Process
Source: J Med Syst. 2024 Feb 14;48(1):20. doi: 10.1007/s10916-024-02042-6 (PMC10866790; doi:10.1007/s10916-024-02042-6)
Supplement: Supplementary file 3 — Supplementary Material 3 [file 10916_2024_2042_MOESM3_ESM.docx]

**Overview of the tasks that the users performed during the study**

| **No.** | **Task** |
| --- | --- |
| 1 | Start the app and go to the Quick Check. |
| 2 | Choose the field that interests you the most and start the Quick Check. |
| 3 | Answer all questions as truthfully as possible. |
| 4 | Display your result. |
| 5 | Choose one of the given health goals. |
| 6 | Register in the app. |
| 7 | Go to your profile and change your password. |
| 8 | Look around in each of the five sections of the app (bottom of the screen). |
| 9 | Ask the chatbot a question. |
| 10 | Log out. |
| 11 | Restart the app and do the two other Quick Checks. |

**Average time needed to complete each individual user task**

| **Task** | **Average time [min:sec]** | **Stdv. [min:sec]** |
| --- | --- | --- |
| 1 | 1:18.0 | 0:35.4 |
| 2 | 0:13.2 | 0:09.6 |
| 3 | 1:17.4 | 0:30.6 |
| 4 | 0:09.0 | 0:04.2 |
| 5 | 0:49.2 | 0:34.2 |
| 6 | 0:52.8 | 0:19.8 |
| 7* | 1:11.4 | 0:37.2 |
| 8 | 2:11,4 | 1:19.2 |
| 9 | 0:57.0 | 0:37.8 |
| 10 | 0:18.0 | 0:10.2 |
| 11 | 5:58.8 | 2:27.0 |
| *Two participants were not able to do the task and were excluded from this task. | | |
